# Association of Blood Donor-derived Cell-free DNA Levels With Banff Scores and Histopathological Lesions in Kidney Allograft Biopsies: Results From an Observational Study

**DOI:** 10.1097/TXD.0000000000001794

**Published:** 2025-04-10

**Authors:** Aylin Akifova, Klemens Budde, Mira Choi, Kerstin Amann, Maike Buettner-Herold, Michael Oellerich, Julia Beck, Kirsten Bornemann-Kolatzki, Ekkehard Schütz, Friederike Bachmann, Fabian Halleck, Eva V. Schrezenmeier, Evelyn Seelow, Bianca Zukunft, Charlotte Hammett, Nathan A. Pohl, Benedetta Mordà, Jan Kowald, Nils Lachmann, Diana Stauch, Bilgin Osmanodja

**Affiliations:** 1 Department of Nephrology and Intensive Care, Charité—Universitätsmedizin Berlin, Berlin, Germany.; 2 Department of Nephropathology, Institute of Pathology, University Hospital Erlangen, Friedrich-Alexander University Erlangen-Nuremberg, Erlangen, Germany.; 3 Department of Clinical Pharmacology, University Medical Center Göttingen, Göttingen, Germany; 4 Chronix Biomedical GmbH, Göttingen, Germany.; 5 Nephrology Unit, University Hospital of Parma, Parma, Italy.; 6 Department of Medicine and Surgery, University of Parma, Parma, Italy.; 7 Medical Department III, Division of Nephrology, University of Leipzig Medical Center, Leipzig, Germany.; 8 Institute for Transfusion Medicine, Histocompatibility and Immunogenetics Laboratory, Charité—Universitätsmedizin Berlin, Berlin, Germany.

## Abstract

**Background.:**

Donor-derived cell-free DNA (dd-cfDNA) is an emerging biomarker of kidney allograft injury, mainly investigated in the context of rejection. However, the dd-cfDNA dynamics in other graft pathologies merit further investigation.

**Methods.:**

In this single-center observational study, we prospectively collected dd-cfDNA at indication biopsies. To evaluate the association between dd-cfDNA and different histological patterns, we correlated absolute and relative dd-cfDNA (thresholds of 50 copies/mL and 0.5%, respectively) with the Banff 2022 lesion scores and the assigned diagnoses.

**Results.:**

We examined 151 dd-cfDNA paired biopsies in 131 kidney transplant recipients and found significantly higher absolute dd-cfDNA levels in antibody-mediated rejection (n, median, IQR: 45, 63 copies/mL, 42–89), microvascular inflammation (MVI) without donor-specific antibodies or C4d-deposition (6, 102 copies/mL, 61–134), mixed rejection (8, 140 copies/mL, 77–171), and BK virus–associated nephropathy (6, 213 copies/mL, 83–298) compared with glomerulonephritis (20, 12 copies/mL, 8–18), calcineurin toxicity (19, 10 copies/mL, 7–16), interstitial fibrosis/tubular atrophy (12, 10 copies/mL, 9–16) and normal histology (6, 9 copies/mL, 7–16). In the multivariable analysis, absolute and relative dd-cfDNA correlated with the peritubular capillaritis (ptc), glomerulitis (g), and tubulitis (t) scores. In the receiver operating characteristic analysis, absolute dd-cfDNA showed best discrimination for MVI of any cause (area under the curve [AUC] 0.88, sensitivity 0.71, specificity 0.86, positive predictive value [PPV] 0.76, negative predictive value [NPV] 0.82), followed by antibody-mediated rejection including mixed rejection (AUC 0.85, sensitivity 0.72, specificity 0.83, PPV 0.69, NPV 0.84), and overall rejection (AUC 0.83, sensitivity 0.66, specificity 0.85, PPV 0.76, NPV 0.77). T cell–mediated rejection was only detectable by dd-cfDNA when associated with vascular lesions.

**Conclusions.:**

Altogether, we conclude that dd-cfDNA-release is not limited to rejection-related injury phenotypes and is mainly driven by MVI in kidney allografts.

## Graphical abstract

**Figure:**
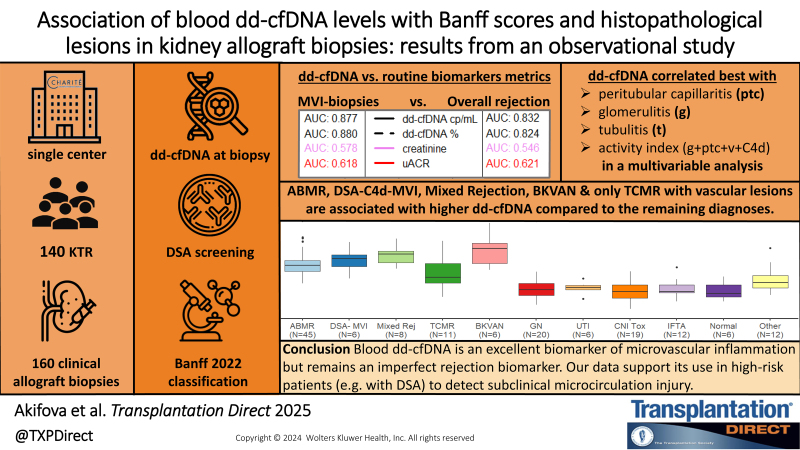

In the era of personalized medicine, numerous biomarkers have emerged to provide more informative monitoring after solid organ transplantation and hereby improve graft survival.^1^ Many promising candidates are being investigated; however, there are some crucial validation steps before implementing biomarkers for real-world application. Ideally, a biomarker should be easy to obtain, at acceptable cost, and highly informative with good overall accuracy (for the particular purpose) to advocate its transition into clinical practice.^2-5^

Recently, the noninvasive injury biomarker donor-derived cell-free DNA (dd-cfDNA) has gained particular interest in the transplant community since early evidence showed promising results supporting further clinical validation and integration of this test.^6^ In brief, cfDNA refers to circulating fragments of nucleic acids that can be captured in peripheral blood resulting from cellular damage processes, such as apoptosis or necrosis.^7,8^ Although low amounts are physiological, excessive cfDNA release indicates abnormal cellular damage because of various underlying conditions.^7,8^ Because novel techniques allow the detection of allogeneic cell-free DNA, this method certainly gained some relevance in kidney transplantation. Detecting fragments of graft-derived DNA in recipients’ blood is based on genetic differences between the donor and recipient; however, the test can be processed without requiring donor and recipient genotypes, making it widely applicable.^9-12^ In addition, its short half-life of 0.5–2 h and injury-dependent release are advantageous and enhance its discriminatory potential for detecting graft injury (eg, because of rejection) compared with biomarkers such as serum creatinine and urinary protein excretion, which reflect graft function and structural integrity of the glomerular barrier instead of actual injury.^8^

Most importantly, dd-cfDNA showed the best diagnostic performance in detecting antibody-mediated rejection (AMR) and mixed rejection, followed by moderate performance in detecting T cell–mediated rejection (TCMR) and borderline changes in previous studies.^13-17^ Unfortunately, the majority of earlier studies only focused on the ability of dd-cfDNA to discriminate rejection but did not specify the type and stage of rejection nor provided insights on the co-occurrence of concurrent lesions or conditions, which might have interfered with the dd-cfDNA results. Although rejection is a leading cause of graft failure, various other pathologies and risk factors determine the posttransplant outcomes in kidney transplant recipients (KTRs).^18,19^ Until now, there is only sparse or controversial evidence on the dynamics of dd-cfDNA in other common pathologies beyond rejection. These include BK virus–associated nephropathy (BKVAN), recurrent glomerular diseases such as recurrent IgA nephropathy (rIgAN) and focal segmental glomerular sclerosis, graft pathologies due to infections, or more rare diagnoses such as deposition disorders and other conditions, where graft function or integrity can be affected (e.g., malignancy, systemic infections).^20-25^

This, along with other questions around this biomarker, such as the optimal intervention thresholds, appropriate context of use, indication and frequency of testing, and the cost-effectiveness of this test, are some crucial aspects that remain subject to ongoing discussions.^26-28^ Considering this, there is an unmet need to gain a more granular understanding of the biological background of dd-cfDNA release and the potential factors that might influence its interpretation before this test can be applied to inform clinical decisions in daily practice.^29^ In respect of the complexity of kidney allograft pathologies and assuming that dd-cfDNA release is not limited to alloimmune-mediated injury, we aimed to assess both absolute and relative values of dd-cfDNA in diverse histopathological patterns, the correlation of dd-cfDNA with Banff lesion scores, and the recently suggested Banff-based activity and chronicity indices in consecutive cases of KTRs undergoing indication biopsies.^30,31^

## Materials and Methods

### Study Design

This single-center, prospective observational study was conducted from August 2020 to July 2024 at the Department of Nephrology and Medical Intensive Care, Charité Universitätsmedizin Berlin (Germany) and approved by the local ethics committee (EA2/144/20). This study was conducted in accordance with the principles of the Declaration of Helsinki and the Declaration of Istanbul as outlined in the ‘Declaration of Istanbul on Organ Trafficking and Transplant Tourism’. All study participants provided written informed consent before any study-related investigation.

### Study Cohort

**FIGURE 1.**
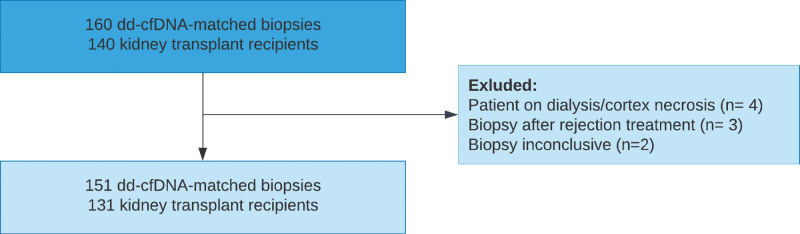
Patient flow diagram. dd-cfDNA, donor-derived cell-free DNA.

For this analysis, we identified all dd-cfDNA-paired biopsies in KTRs undergoing indication biopsy because of acute or chronic graft dysfunction. Increase of serum creatinine, persistence of anti-HLA donor-specific antibodies (DSAs), or proteinuria were the most common biopsy indications. Biopsies with incomplete Banff scoring in the pathology reports were excluded from the final analysis. In addition, dd-cfDNA samples obtained up to 3 mo after antirejection treatment were excluded. Detailed eligibility criteria are listed in Table 1. The screening process is outlined in Figure 1.

**TABLE 1.** Eligibility criteria for enrollment of study participants

Inclusion criteria • Patients after kidney transplantation, who underwent clinical indication biopsy • Functioning kidney graft, at least 2 wk after last transplantation • Patients 18 y or older • Patients provided written informed consent
Exclusion criteria • Inadequate histological samples (not representative enough to assign diagnosis using the Banff coding system) • Patients unable to provide written informed consent • Pregnant or breastfeeding persons • Patients with multiorgan transplantation • Patients with dd-cfDNA collection after antirejection therapy was applied

dd-cfDNA, donor-derived cell-free DNA.

### Diagnostic Procedures

#### dd-cfDNA Measurements

For each dd-cfDNA measurement,^9^ up to 16 mL of blood was drawn into 2 certified blood collection tubes (Streck Corp., Omaha, NB) at the time of indication biopsy and shipped to the processing laboratory at ambient temperature within 3 d after sample collection. No specimens were collected within 48 h after kidney allograft biopsy to prevent dd-cfDNA release from the biopsy.^32^ Additionally, dd-cfDNA was isolated and quantified using the droplet digital polymerase chain reaction method, providing both relative and absolute values of dd-cfDNA, with cutoffs of 0.5% and 50 copies/mL, respectively, as established previously.^10^ Detailed methods of dd-cfDNA processing are outlined in SDC, Materials and Methods.

### Detection of Anti-HLA DSAs

Assessment of anti-HLA DSAs was performed using Luminex-based LABScreen SAB assay (One Lambda, Canoga Park, CA), as widely standardized.^33^ Standard-of-care CDC-crossmatch testing was performed in all subjects pretransplant and de novo DSA (dnDSA) were detected by routine annual screening by LABScreen Mixed and SAB assay posttransplant.^34^ In general, dnDSA were considered positive above a mean fluorescence intensity (MFI) threshold of 1000, also considering other factors such as plausibility.^34,35^ If several beads with different alleles of the donor antigen are present in the test, the MFI of the highest bead value was used if the exact donor allele was not known. Three MFI values were assessed: (1) at the first occurrence of DSA, (2) the MFI_max_ over time, and (3) in the most recent test before biopsy.

### Histological Assessment of Kidney Allograft Biopsies

Kidney biopsy samples were obtained by ultrasound-guided, and in 2 cases by computed tomography–guided needle biopsy and were formalin-fixed and embedded in paraffin for routine workup including light microscopy, immunohistochemistry and in a subgroup (77/151) electron microscopy. Histological data were retrieved from the histopathology reports, which were based on the current Banff classifications at the time of biopsy (either 2019 or 2022). A final diagnosis was assigned for each biopsy by 2 clinicians (A.A. and B.O.) in consensus after including clinical data (eg, DSA status, calcineurin inhibitor [CNI] dosage, transcriptome analysis) according to the Banff 2022 classification.^36^

### Statistical Methods

The demographics of the study participants were summarized as median and interquartile range (IQR) for continuous covariates (or as mean ± SD for normally distributed parameters), and absolute and relative frequencies for categorical parameters. The Mann-Whitney U test was used to detect differences in nonparametric continuous variables between 2 independent groups. For >2 independent groups, the Kruskal-Wallis test with Dunn post hoc test and Holm correction for multiple comparisons was used. Diagnostic test metrics of dd-cfDNA were assessed using receiver operating characteristic (ROC) analysis with prespecified thresholds (50 copies/mL for absolute and 0.5% for relative dd-cfDNA) and compared with those of urine albumin–creatinine ratio (uACR) with prespecified cutoffs of 300 and 1000 mg/g, as well as creatinine for all patients with kidney allograft biopsy as the gold standard and compared using the method by DeLong. For dd-cfDNA, optimal cutoffs based on our data were calculated using the Youden method. The correlation analysis was performed using the Spearman correlation test. Statistical tests were 2-tailed and a *P* value <0.05 was considered statistically significant. Data analyses were performed using R, version 4.3.0 (The R Foundation for Statistical Computing, Vienna, Austria).

## RESULTS

### Study Population

In total, we identified 160 dd-cfDNA-paired biopsies in 140 KTR who underwent an indication allograft biopsy between August 2020 and July 2024. From these, 9 dd-cfDNA biopsy matches were excluded because of cortical necrosis (n = 4), incomplete Banff scoring (n = 2), or dd-cfDNA collection after antirejection treatment (n = 3) (Figure 1).

Finally, 151 dd-cfDNA-matched biopsies from 131 KTR were included in the analysis. Detailed patient demographics and baseline characteristics are summarized in Table 2. The overall biopsy-proven rejection rate was 64 (42.4%) of 151, of which 45 (70.3%) of 64 rejection biopsies showed AMR (active AMR = 8, chronic active AMR = 36, probable AMR = 1), 11 of 64 biopsies showed TCMR excluding borderline changes (18%) (TCMR IA = 6, TCMR IB = 2, TCMR IIB = 2, caTCMR = 1) and 8 (13%) of 64 biopsies showed signs of both AMR and TCMR (mixed rejection).

**TABLE 2.** Baseline characteristics of patients with dd-cfDNA biopsy matches included in the analysis

Baseline characteristics	Patients/transplants	Biopsies
N	131	151
Demographics		
Age at transplant, y	45.5 (32.6–55.3)	—
Sex female/male	29% (38)/71% (93)	—
Cause of kidney failure		
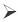 IgA nephropathy	22.1% (29)	—
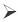 Reflux nephropathy	15.3% (20)	—
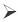 Unknown	14.5% (19)	—
ADPKD	13% (17)	—
Systemic autoimmune	8.4% (11)	—
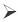 FSGS	6.9% (9)	—
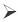 Alport	4.6% (6)	—
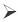 Hypertensive/diabetic sclerosis	3.1% (4)	—
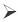 Other genetic/congenital	5.3% (7)	—
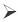 Other	6.9% (9)	—
Kidney replacement therapy		
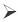 HD	66.4% (87)	—
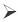 PD	13.7% (18)	—
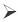 HD after PD	3.1% (4)	—
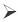 Preemptive transplantation	14.5% (19)	—
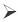 Unknown	2.3% (3)	—
Transplantation		
Living donation	53.4% (70)	—
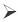 Related donor	54.3% (38)	—
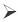 AB0 incompatible	22.9% (16)	—
HLA mismatch grade	3 (2–4)	
Induction IS		
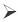 Basiliximab	84.7% (111)	—
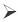 ATG	8.4% (11)	—
Maintenance IS at biopsy	—	
CNI-based IS	—	92.1% (139)
Tacrolimus	—	82.0% (114)
Ciclosporin	—	18.0% (25)
Belatacept	—	3.3% (5)
Mycophenolic acid	—	90.7% (131)
Steroid	—	64.9% (98)
DSA	52.7% (69)	—
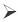 Preformed DSA	10.1% (7)	—
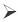 Preformed and de novo DSA	4.3% (3)	—
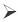 De novo DSA	85.5% (59)	—
Transplant age, y	—	7.5 (1.6–13.7)
Marginal biopsy adequacy	—	87% (131)
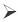 Glomerular count	—	11 (8–16)
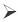 Electron microscopy performed	—	51% (77)
Graft function at biopsy		
Creatinine at biopsy (mg/dL)	—	2.25 (1.68–2.94)
Urine albumin–creatinine ratio (mg/g)	—	180 (34–763)

Values are shown as median (interquartile range) or relative and absolute frequency.

ADPKD, autosomal dominant polycystic disease; ATG, anti-thymocyte globulin; CNI, calcineurin inhibitor; DSA, donor-specific anti-HLA antibody; FSGS, focal segmental glomerular sclerosis; HD, hemodialysis; IS, immunosuppression; PD, peritoneal dialysis

Nonrejection biopsies showed the following diagnoses: recurrent or de novo glomerulonephritis (n = 20; 13.3%), CNI-related toxicity changes (n = 19; 12.6%), interstitial fibrosis/tubular atrophy (IFTA) (n = 12; 8%), microvascular inflammation DSA and C4d (DSA-MVI) (n = 6; 4%), BKVAN (n = 6; 4%), ascending urinary tract infection (UTI) (n = 6; 4%), and normal, referring to biopsies with unremarkable histological findings (n = 6; 4%). Finally, biopsies that showed acute tubular necrosis with unspecific changes (n = 8), tubulointerstitial nephritis (TIN) not related to TCMR, BKVAN, or UTI (n = 2), and 2 cases of renal graft amyloidosis were classified as “Other” (n = 12; 8%).

Among all study participants, 69 (52.7%) patients had serological evidence of DSA at time of biopsy, of which 7 (10.1%) had preexisting DSA that persisted or reappeared after transplantation, 3 (4.3%) had preexisting and dnDSA, and 59 (85.5%) had dnDSA, the latter of which developed after a median of 3.8 years (IQR 1.4–7.0 y) posttransplant.

### Comparison of dd-cfDNA in Different Histopathological Diagnoses

Overall, 55 (36.4%) of 151 samples had elevated absolute dd-cfDNA levels (≥50 copies/mL). AMR, DSA-MVI, and mixed rejection accounted for 42 (76.4%) of 55 of the dd-cfDNA-positive samples, whereas TCMR and BKVAN accounted for 4 (7.3%) of 55 and 5 (9.1%) of 55 of the positive samples, respectively.

**FIGURE 2.**
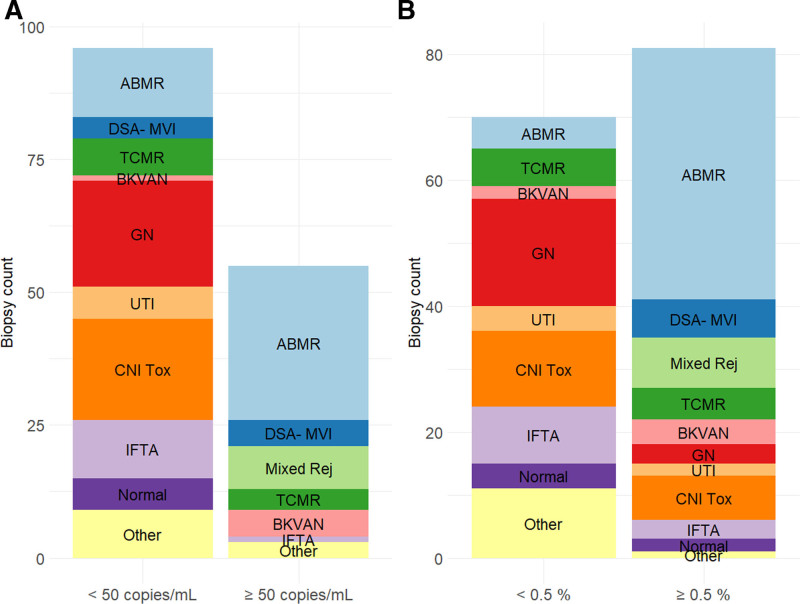
Frequency of histopathological diagnoses grouped by the prespecified thresholds for absolute dd-cfDNA of 50 copies/mL (A), and relative dd-cfDNA of 0.5% (B). AMR, antibody-mediated rejection; BKVAN, BK virus–associated nephropathy; CNI Tox, calcineurin inhibitor toxicity; dd-cfDNA, donor-derived cell-free DNA; DSA- MVI, DSA-negative microvascular inflammation; GN, glomerulonephritis; IFTA, interstitial fibrosis/tubular atrophy; Mixed Rej, mixed rejection; TCMR, T cell–mediated rejection; TIN, tubulointerstitial nephritis; UTI, urinary tract infection.

Importantly. we noted that a higher number of samples (81/151 [53.6%]) had increased relative dd-cfDNA levels (≥0.5% of the total cfDNA). With relative quantification, AMR, DSA-MVI, and mixed rejection accounted for 54 (66.7%) of 81 of dd-cfDNA positive samples. However, a significant proportion of samples with normal histology (6/81, 7.4%), IFTA (12/81, 14.8%), and CNI toxicity (19/81, 23.5%) showed elevated relative dd-cfDNA. The frequencies of histological diagnoses above and below the predefined thresholds are illustrated in Figure 2A and B for absolute and relative dd-cfDNA, respectively.

**FIGURE 3.**
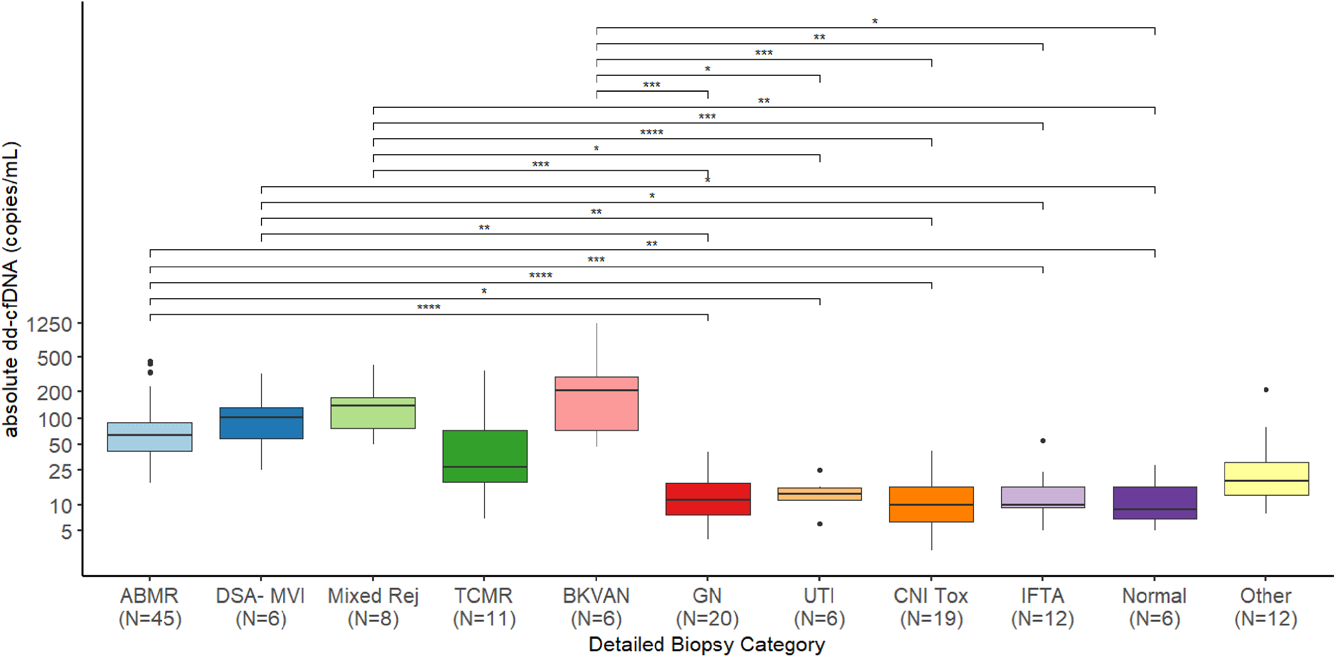
Donor-derived cell-free DNA measured as copies/mL depending on biopsy results. Significant differences between groups are highlighted with asterisks. AMR, antibody-mediated rejection; BKVAN, BK virus–associated nephropathy; CNI Tox, calcineurin inhibitor toxicity; dd-cfDNA, donor-derived cell-free DNA; DSA- MVI, DSA-negative microvascular inflammation; GN, glomerulonephritis; IFTA, interstitial fibrosis/tubular atrophy; Mixed Rej, mixed rejection; TCMR, T cell–mediated rejection; TIN, tubulointerstitial nephritis; UTI, urinary tract infection.

Exploring the associations between dd-cfDNA and certain histological diagnoses, we found that absolute dd-cfDNA was higher in ABMR (median 63 copies/mL, IQR 42–89), DSA-MVI (median 102 copies/mL, IQR 61–134), mixed rejection (median 140 copies/mL, IQR 77–171), and BKVAN (median 213 copies/mL, IQR 83–298) compared with glomerulonephritis (GN) (median 12 copies/mL, IQR 8–18), CNI toxicity (median 10 copies/mL, IQR 7–16), IFTA (median 10, IQR 9–16), and normal histology (median 9, IQR 7–16). Additionally, patients with AMR, mixed rejection, and BKVAN had higher absolute dd-cfDNA levels than patients with UTI (median 14, IQR 12–16) (Figure 3).

**FIGURE 4.**
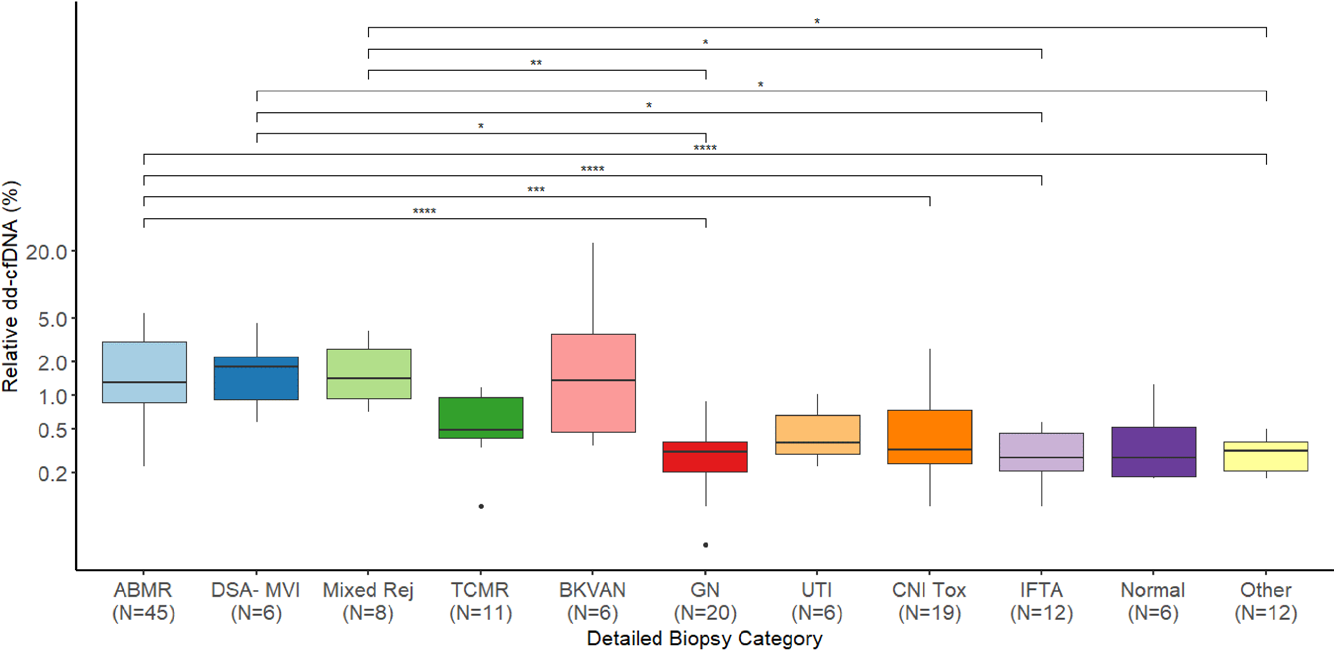
Donor-derived cell-free DNA measured as % of total cfDNA depending on biopsy results. Significant differences between groups are highlighted with asterisks. AMR, antibody-mediated rejection; BKVAN, BK virus–associated nephropathy; CNI Tox, calcineurin inhibitor toxicity; dd-cfDNA, donor-derived cell-free DNA; DSA- MVI, DSA-negative microvascular inflammation; GN, glomerulonephritis; IFTA, interstitial fibrosis/tubular atrophy; Mixed Rej, mixed rejection; TCMR, T cell–mediated rejection; TIN, tubulointerstitial nephritis; UTI, urinary tract infection.

Comparably, relative dd-cfDNA was significantly higher in AMR (median 1.31%, IQR 0.87–3.00), DSA-MVI (median 1.83%, IQR 1.01–2.22), and mixed rejection (median 1.43%, IQR 0.95–2.63) in comparison with GN (median 0.31%, IQR 0.21–0.38), IFTA (median 0.28%, IQR 0.21–0.46), and other diagnoses (median 0.32%, IQR 0.21–0.38). Additionally, patients with AMR had higher relative dd-cfDNA levels than patients with CNI toxicity (median 0.32%, IQR 0.25–0.74) (Figure 4).

### Correlation of dd-cfDNA With the Banff Lesion Scores

In the univariable analysis (Spearman correlation), absolute dd-cfDNA levels correlated with Banff lesion scores for glomerulitis (g) (*ρ* = 0.53, *P* < 0.001), peritubular capillaritis (ptc) (*ρ* = 0.62, *P* < 0.001), C4d (*ρ* = 0.33, *P* < 0.001), intimal arteritis (v) (*ρ* = 0.22, *P* = 0.006), interstitial inflammation (i) (*ρ* = 0.29, *P* < 0.001), tubulitis (t) (*ρ* = 0.23, *P* = 0.004), and glomerular basement membrane double contours (cg) (*ρ* = 0.33, *P* < 0.001). In the multivariable analysis (linear regression), log-transformed absolute dd-cfDNA levels correlated with g (B = 0.28, *P* = 0.012), ptc (B = 0.42, *P* < 0.001), v (B = 0.52, *P* = 0.039), and t (B = 0.49, *P* = 0.003) scores (Table 3).

**TABLE 3.** Univariable (Spearman correlation) and multivariable analysis (linear regression) examining the correlation of histopathological Banff scores with log-transformed absolute dd-cfDNA (copies/mL).

Banff scores	Univariable analysis (Spearman correlation)		Multivariable analysis (linear regression)	
	Spearman *ρ*	*P*	Unstandardized coefficient B	*P*
g	0.53	**<0.001**	0.28	**0.01**
ptc	0.62	**<0.001**	0.42	**<0.001**
C4d	0.33	**<0.001**	0.12	0.18
v	0.22	**0.006**	0.52	**0.04**
i	0.29	**<0.001**	0.07	0.71
ti	0.15	0.07	−0.24	0.24
i-IFTA	−0.08	0.31	−0.07	0.63
t-IFTA	−0.02	0.80	−0.04	0.82
t	0.23	**0.004**	0.49	**0.003**
ci	−0.07	0.42	0.45	0.37
ct	−0.05	0.51	−0.46	0.36
cv	−0.09	0.29	−0.09	0.42
cg	0.33	**<0.001**	0.06	0.51
mm	−0.12	0.15	−0.10	0.34
aah	0.13	0.88	−0.01	0.92

Bold values indicate that *P* value <0.05 was considered statistically significant. aah, hyaline arteriolar thickening; cg, glomerular basement membrane double contours; ci; interstitial fibrosis; ct, tubular atrophy; cv, vascular fibrous intimal thickening; g, glomerulitis; i, interstitial inflammation; i-IFTA, inflammation in area of interstitial fibrosis/tubular atrophy; ; mm, mesangial matrix expansion; ptc, peritubular capillaritis; ti, total inflammation; t-IFTA, tubulitis in areas of interstitial fibrosis; v, intimal arteritis.

In the univariable analysis, relative dd-cfDNA levels also correlated with the Banff lesion scores g (*ρ* = 0.52, *P* < 0.001), ptc (*ρ* = 0.57, *P* < 0.001), C4d (*ρ* = 0.34, *P* < 0.001), v (*ρ* = 0.19, *P* = 0.021), and cg (*ρ* = 0.37, *P* < 0.001), but not with t (*ρ* = 0.13, *P* = 0.12) or i (*ρ* = 0.14, *P* = 0.077). In the multivariable analysis, log-transformed relative dd-cfDNA levels correlated with g (B = 0.24, *P* = 0.024), ptc (B = 0.31, *P* = 0.002), and t (B = 0.39, *P* = 0.012) scores (Table 4).

**TABLE 4.** Univariable (Spearman correlation) and multivariable analysis (linear regression) examining the correlation of histopathological Banff scores with log-transformed relative dd-cfDNA (%)

Banff scores	Univariable analysis (Spearman correlation)		Multivariable analysis (linear regression)	
	Spearman *ρ*	*P*	Unstandardized coefficient B	*P*
g	0.52	**<0.001**	0.24	**0.024**
ptc	0.56	**<0.001**	0.31	**0.002**
C4d	0.34	**<0.001**	0.16	0.07
v	0.19	**0.02**	0.30	0.21
i	0.14	0.08	−0.23	0.24
ti	0.12	0.14	−0.05	0.80
i-IFTA	−0.02	0.79	−0.13	0.40
t-IFTA	−0.01	0.86	0.10	0.58
t	0.13	0.12	0.39	**0.012**
ci	-0.01	0.95	0.43	0.37
ct	0.01	0.94	-0.32	0.51
cv	−0.03	0.69	-0.08	0.47
cg	0.37	**<0.001**	0.10	0.20
mm	−0.03	0.69	−0.07	0.48
aah	0.12	0.13	0.07	0.27

Bold values indicate that *P* value <0.05 was considered statistically significant. aah, hyaline arteriolar thickening; cg, glomerular basement membrane double contours; ci; interstitial fibrosis; ct, tubular atrophy; cv, vascular fibrous intimal thickening; g, glomerulitis; i, interstitial inflammation; i-IFTA, inflammation in area of interstitial fibrosis/tubular atrophy; ; mm, mesangial matrix expansion; ptc, peritubular capillaritis; ti, total inflammation; t-IFTA, tubulitis in areas of interstitial fibrosis; v, intimal arteritis.

### Banff Lesions That Independently Correlated With dd-cfDNA

**FIGURE 5.**
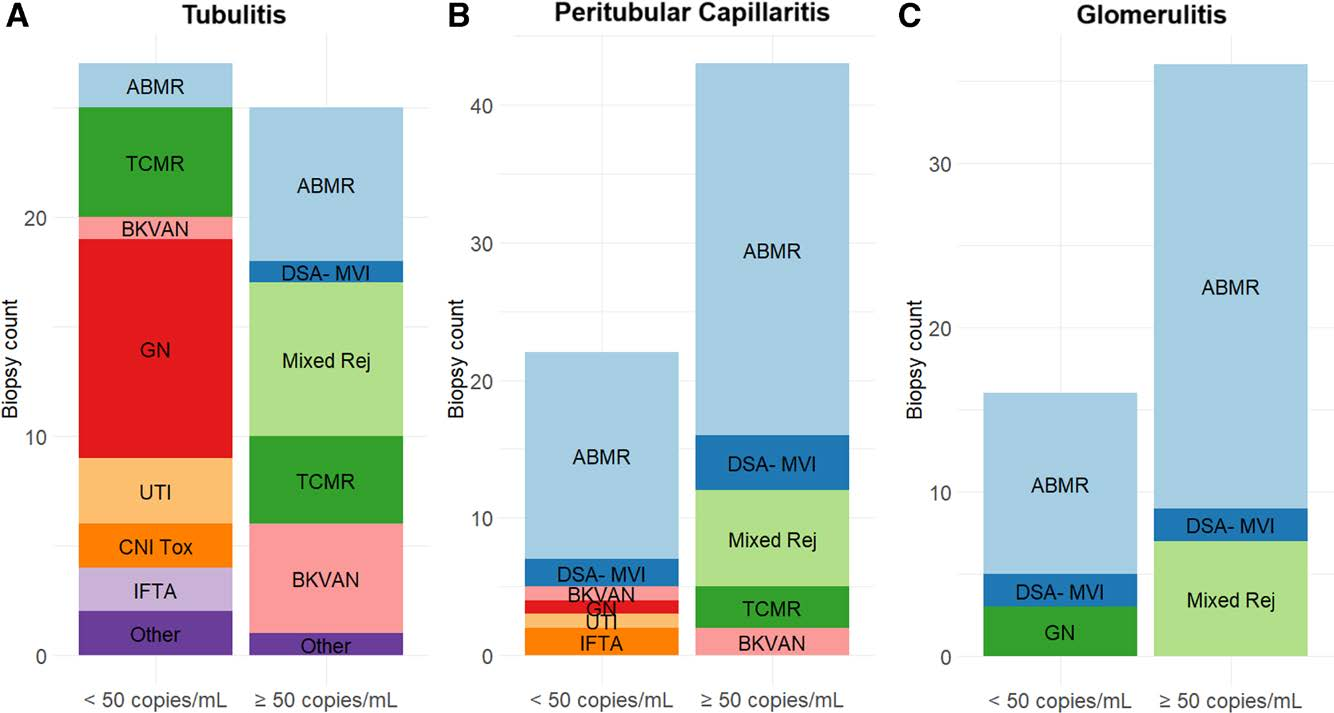
Frequency of histopathological diagnoses grouped by absolute dd-cfDNA in biopsies with tubulitis (t > 0) (A), biopsies with peritubular capillaritis (ptc > 0) (B), and biopsies with glomerulitis (g > 0) (C). dd-cfDNA, donor-derived cell-free DNA; g, glomerulitis; ptc, peritubular capillaritis; t, tubulitis.

Because of their strong correlation with dd-cfDNA, we further analyzed biopsies showing tubulitis, peritubular capillaritis, and glomerulitis. The frequency of diagnosis showing t, ptc, or g lesions grouped by the absolute dd-cfDNA cutoff is summarized in Figure 5. We further described the co-occurrence of other Banff lesions for biopsies showing t (Table 5), ptc (Table 6), and g (Table 7).

**TABLE 5.** Histopathological diagnoses of patients with tubulitis (t > 0) together with absolute and relative dd-cfDNA, and selected Banff scores

Diagnosis	n	Absolute dd-cfDNA (copies/mL)	Relative dd-cfDNA (%)	t score	i score	g score	ptc score	v score
BKVAN	6	213 (83–298)	1.42 (0.53–3.81)	2 (2–3)	3 (1–3)	0 (0–0)	1 (0–2)	0 (0–0)
TCMR	9	30 (22–87)	0.49 (0.40–1.10)	2 (2–3)	2 (2–2)	0 (0–0)	0 (0–1)	0 (0–0)
Mixed Rej.	7	136 (75–156)	1.61 (1.12–2.66)	2 (2–2)	2 (2–2)	2 (1–2)	2 (2–2)	1 (1–1)
DSA− MVI	1	323	0.74	2	2	3	2	0
UTI	3	11–25	0.23–0.79	1–3	1–3	0–0	0–2	0–0
GN	10	12 (8–13)	0.31 (0.22–0.35)	1 (1–2)	1 (1–2)	0 (0–0)	0 (0–0)	0 (0–0)
Other	3	13–214	0.30–0.50	1–2	2-3	0	0	0
IFTA	2	10–24	0.19–0.51	1–2	1	0	0–2	0
AMR	9	81 (60–108)	3.41 (2.70–3.80)	1 (1–1)	1 (1–1)	1 (1–1)	2 (2–2)	0 (0–0)
CNI Tox.	2	6–13	0.29–0.70	1–1	0–0	0–0	0–0	0-0

All values are presented as median (interquartile range) for diagnoses with >4 values, or minimum–maximum for diagnoses with 4 or less values.

AMR, antibody-mediated rejection; BKVAN, BK virus–associated nephropathy; CNI Tox., calcineurin inhibitor toxicity; dd-cfDNA, donor-derived cell-free DNA; DSA− MVI, DSA-negative microvascular inflammation; g, glomerulitis; GN, glomerulonephritis; i, interstitial inflammation; IFTA, interstitial fibrosis/tubular atrophy; ptc, peritubular capillaritis; Mixed Rej., mixed rejection; TCMR, T cell-mediated rejection; UTI, urinary tract infection; v, intimal arteritis.

**TABLE 6.** Histopathological diagnoses of patients with peritubular capillaritis (ptc > 0) together with absolute and relative dd-cfDNA, and selected Banff scores

Diagnosis	n	Absolute dd-cfDNA (copies/mL)	Relative dd-cfDNA (%)	t score	i score	g score	ptc score	v score
Mixed Rej.	7	136 (75–161)	1.61 (1.06–2.66)	2 (2–2)	2 (1–2)	2 (1–2)	2 (2–2)	1 (1–1)
DSA− MVI	6	102 (61–134)	1.83 (1.01–2.22)	0 (0–0)	1 (0–1)	2 (1–3)	2 (2–2)	0 (0–0)
AMR	42	62 (42–89)	1.34 (0.88–2.97)	0 (0–0)	0 (0–1)	1 (1–2)	2 (2–2)	0 (0–0)
BKVAN	3	46–1228	0.35–23.71	2–3	1–3	0–0	1–3	0–0
TCMR	3	60–351	0.49–1.18	2–3	2–2	0–0	1–3	0–0
IFTA	2	10–15	0.19–0.31	0–2	0–1	0	1–2	0
UTI	1	16	0.23	2	3	0	2	0
GN	1	11	0.32	1	1	0	1	0

All values are presented as median (interquartile range) for diagnoses with >4 values, or minimum–maximum for diagnoses with 4 or less values.

AMR, antibody-mediated rejection; BKVAN, BK virus–associated nephropathy; CNI Tox., calcineurin inhibitor toxicity; dd-cfDNA, donor-derived cell-free DNA; DSA− MVI, DSA-negative microvascular inflammation; g, glomerulitis; GN, glomerulonephritis; i, interstitial inflammation; IFTA, interstitial fibrosis/tubular atrophy; ptc, peritubular capillaritis; Mixed Rej., mixed rejection; TCMR, T cell-mediated rejection; UTI, urinary tract infection; v, intimal arteritis.

**TABLE 7.** Histopathological diagnoses of patients with glomerulitis (g > 0) together with absolute and relative dd-cfDNA, and selected Banff scores

Diagnosis	n	Absolute dd-cfDNA (copies/mL)	Relative dd-cfDNA (%)	t score	i score	g score	ptc score	v score
DSA− MVI	4	77 (43–160)	1.83 (1.54–1.98)	0 (0–1)	1 (1–1)	3 (2–3)	2 (2–2)	0 (0–0)
Mixed Rej.	7	144 (108–173)	1.61 (1.12–2.66)	2 (1–2)	2 (2–2)	2 (2–2)	2 (2–2)	1 (1–1)
AMR	38	65 (44–89)	1.54 (0.81–3.30)	0 (0–0)	1 (0–1)	2 (1–3)	2 (2–2)	0 (0–0)
GN	3	13–34	0.30–0.89	0–1	0–1	1–1	0–0	0–0

All values are presented as median (interquartile range) for diagnoses with >4 values, or minimum-maximum for diagnoses with 4 or less values^.^

AMR, antibody-mediated rejection; dd-cfDNA, donor-derived cell-free DNA; DSA− MVI, DSA-negative microvascular inflammation; g, glomerulitis; GN, glomerulonephritis; i, interstitial inflammation; Mixed Rej., mixed rejection; v, intimal arteritis.

Of the 52 biopsy samples with tubulitis (t > 0), 6 (11.5%) showed t3, 21 (40.4%) showed t2, and 25 (48.1%) showed t1. Concomitant interstitial inflammation (i > 0) was present in 46 biopsies (88.5%), glomerulitis (g > 0) in 17 (32.7%), peritubular capillaritis (ptc > 0) in 24 (46.2%), and intimal arteritis (v > 0) in 7 (13.5%). Among biopsies with tubulitis, only those with AMR, mixed rejection, DSA-MVI, and BKVAN had a median absolute dd-cfDNA above the prespecified cutoff of 50 copies/mL. Additionally, 1 of 2 biopsies with tubulointerstitial nephritis (tubulointerstitial inflammation without TCMR diagnosis and exclusion of UTI), which were classified as “Other” had absolute dd-cfDNA above the cutoff.

Of the 65 biopsy samples with peritubular capillaritis (ptc > 0), 10 (15.4%) showed ptc3, 42 (64.6%) showed ptc2, and 13 (20%) showed ptc1. Concomitant glomerulitis (g > 0) was present in 46 biopsies (70.8%), tubulitis (t > 0) in 24 (36.9%), interstitial inflammation (i > 0) in 41 (63.1%), and intimal arteritis (v > 0) in 5 (7.7%). Most biopsies with peritubular capillaritis were diagnosed as AMR (42/65, 64.6%), DSA-MVI (6/65, 9.2%), or mixed rejection (7/65, 10.8%).

Of the 52 biopsy samples with glomerulitis (g > 0), 14 (26.9%) showed g3, 14 (26.9%) showed g2, and 24 (46.2%) showed g1. Concomitant peritubular capillaritis (ptc > 0) was present in 46 biopsies (88.5%), tubulitis (t > 0) in 17 (32.7%), interstitial inflammation (i > 0) in 31 (59.6%), and intimal arteritis (v > 0) in 6 (11.5%). Most biopsies with glomerulitis were diagnosed as ABMR (38/52, 73.1%), DSA-MVI (4/52, 7.7%) or mixed rejection (7/52, 13.5%).

### Correlation of dd-cfDNA With the Banff-based Activity and Chronicity Indices

Finally, we correlated the dd-cfDNA levels with Banff-based MVI index (g+ptc), activity index (AI; g+ptc+C4d+v), and chronicity index (CI; ci+ct+cv+cg*2). Absolute dd-cfDNA was strongly correlated with the MVI index (ρ = 0.65, *P* < 0.001) and AI (ρ = 0.67, *P* < 0.001), but only weakly correlated with the CI (ρ = 0.16, *P* = 0.048). Relative dd-cfDNA also was strongly correlated with the MVI index (ρ = 0.60, *P* < 0.001) and AI (ρ = 0.63, *P* < 0.001), but only weakly correlated with the CI (ρ = 0.23, *P* = 0.004) (Table 8).

**TABLE 8.** Correlation of absolute and relative dd-cfDNA and indices based on Banff scores

	Absolute dd-cfDNA (copies/mL)		Relative dd-cfDNA (%)	
	Spearman *ρ*	*P*	Spearman *ρ*	*P*
MVI index (g+ptc)	0.65	**<0.001**	0.60	**<0.001**
Activity index (g+ptc+C4d+v)	0.67	**<0.001**	0.63	**<0.001**
Chronicity index (ci+ct+cv+2*cg)	0.16	**0.048**	0.23	**0.004**

Bold values indicate that *P* value <0.05 was considered statistically significant. cg, glomerular basement membrane double contours; ci; interstitial fibrosis; ct, tubular atrophy; cv, vascular fibrous intimal thickening; dd-cfDNA, donor-derived cell-free DNA; g, glomerulitis; MVI, microvascular inflammation; ptc, peritubular capillaritis; v, intimal arteritis.

### Diagnostic Metrics of dd-cfDNA in Rejection Versus Nonrejection Biopsies

**FIGURE 6.**
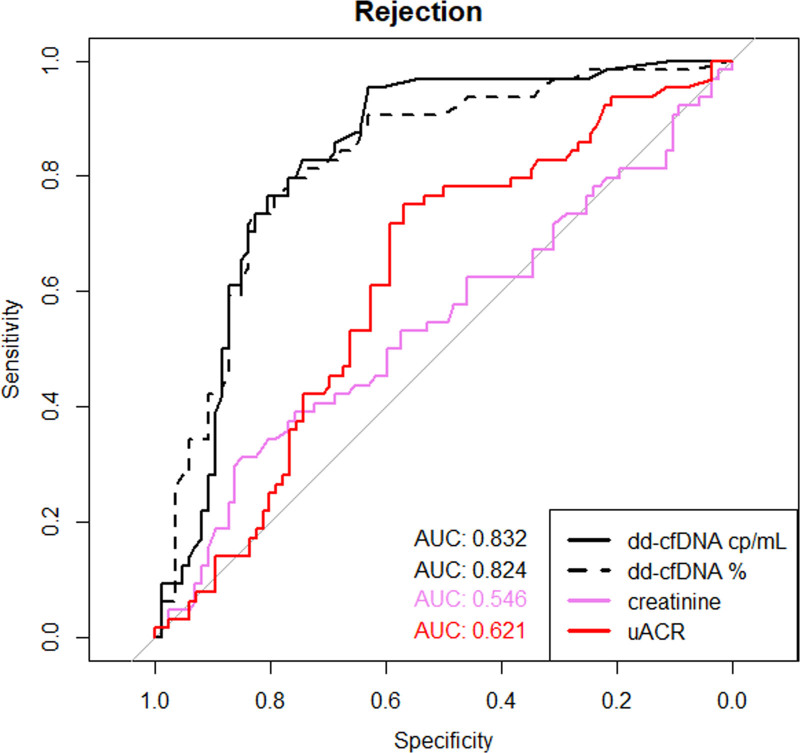
Comparison of the AUC of the receiver-operating characteristics of absolute dd-cfDNA copies/mL, relative dd-cfDNA (%), creatinine, and uACR for the discrimination of rejection vs no rejection. AUC, area under the curve; dd-cfDNA, donor-derived cell-free DNA; uACR, urine albumin–creatinine ratio.

**FIGURE 7.**
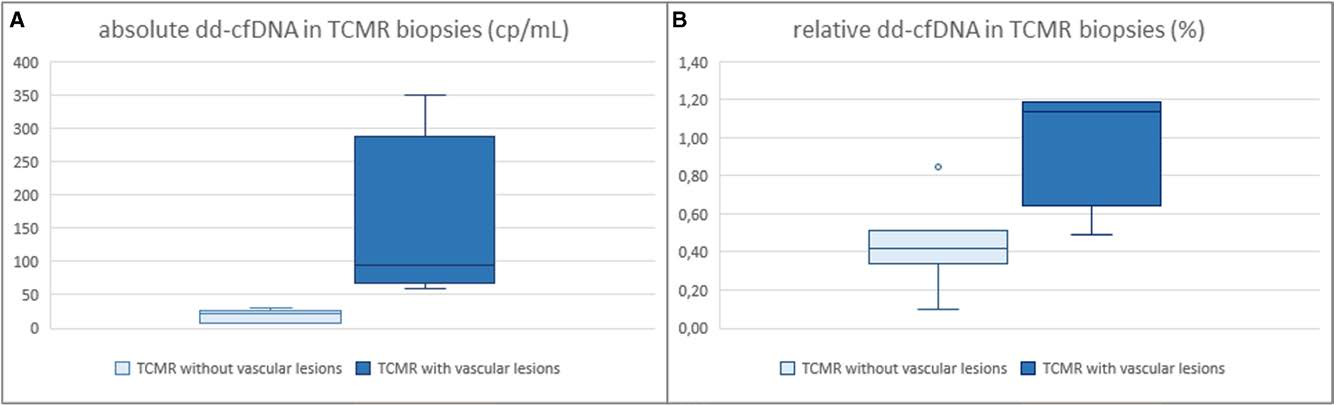
Box plots showing absolute dd-cfDNA, median 27 copies/mL (18.5–73.5) (A) and relative dd-cfDNA (B), median 0.49% (0.41–0.98) in TCMR biopsies presenting with vascular lesions according to the Banff classification (n = 4, ptc>0, v > 0) and without vascular lesions (n = 7). dd-cfDNA, donor-derived cell-free DNA; ptc, peritubular capillaritis; TCMR, T cell–mediated rejection.

We performed ROC analysis to determine the diagnostic test metrics of dd-cfDNA and routine biomarkers to detect rejection (AMR, TCMR, and mixed rejection) using the prespecified cutoffs (50 copies/mL for absolute dd-cfDNA, and 0.5% for relative dd-cfDNA), and an optimal decision threshold based on the Youden method. The main results are summarized in Figure 6 and Table 9. The area under the curve (AUC)-ROC of both absolute (0.83) and relative (0.82) dd-cfDNA were higher than the AUC-ROC of uACR (0.62, with lower values no rejection) and creatinine (0.56, with higher values indicating no rejection) (all pairwise comparisons with *P* < 0.001). Although both absolute and relative dd-cfDNA discriminated AMR, including mixed rejection from the remaining diagnoses with an AUC of 0.85 (test metrics in Table 9), dd-cfDNA did not discriminate pure TCMR from the remaining diagnoses (absolute, AUC 0.53; relative, AUC 0.52). Nevertheless, if TCMR was associated with vascular lesions (g, ptc, or v > 0), the dd-cfDNA values were found to be above the predefined thresholds (Figure 7).

**TABLE 9.** Diagnostic test metrics for the discrimination of rejection from nonrejection biopsies, AMR from non-ABR, and MVI from non-MVI biopsies using absolute and relative dd-cfDNA with prespecified and optimal decision thresholds

Status	AUC	Threshold	Sensitivity, %	Specificity, %	NPV, %	PPV, %
Rejection/abs. dd-cfDNA	0.83	50 copies/mL	66	85	77	76
Rejection/abs. dd-cfDNA	0.83	17.5 copies/mL	95	63	95	66
Rejection/rel. dd-cfDNA	0.82	0.5%	83	68	84	65
Rejection/rel. dd-cfDNA	0.82	0.64%	77	80	72	84
AMR + mixed rejection/abs. dd-cfDNA	0.85	50 copies/mL	72%	83	84	69
AMR + mixed rejection/rel. dd-cfDNA	0.85	0.5 %	90	66	93	59
MVI/abs. dd-cfDNA	0.88	50 copies/mL	71	86	82	76
MVI/abs. dd-cfDNA	0.88	37.5 copies/mL	85	83	89	76
MVI/rel. dd-cfDNA	0.88	0.50%	92	68	93	67
MVI/rel. dd-cfDNA	0.88	0.55%	92	77	93	72

AMR, antibody-mediated rejection; AUC, area under the curve; dd-cfDNA, donor-derived cell-free DNA; MVI, microvascular inflammation; NPV, negative predictive value; PPV, positive predictive value.

### Diagnostic Metrics of dd-cfDNA in Biopsies With MVI Versus No MVI

**FIGURE 8.**
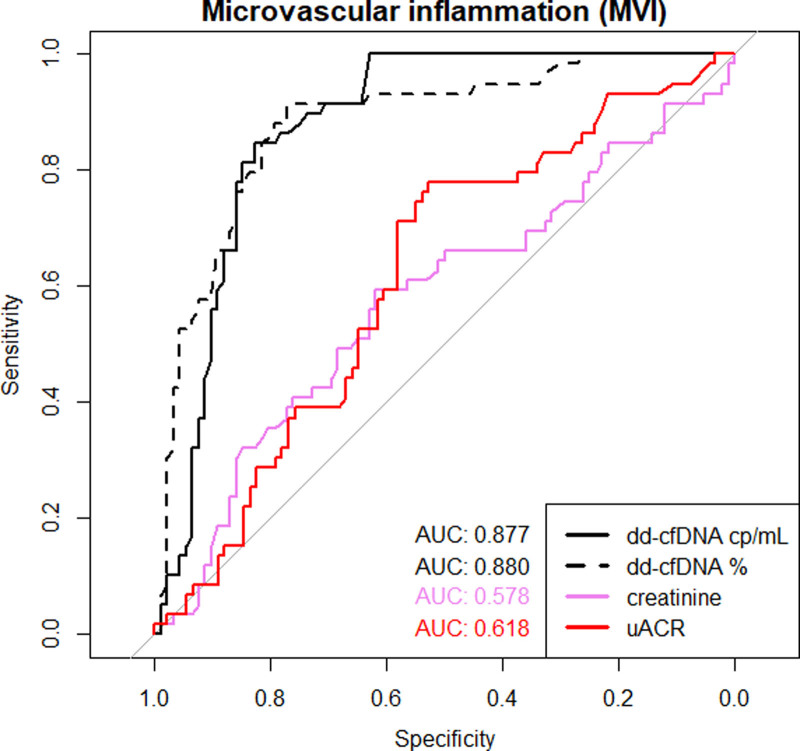
Comparison of the AUC of the receiver-operating characteristics of absolute dd-cfDNA copies/mL, relative dd-cfDNA (%), creatinine, and uACR for the discrimination of MVI vs no MVI. AUC, area under the curve; dd-cfDNA, donor-derived cell-free DNA; MVI, microvascular inflammation; uACR, urine albumin–creatinine ratio.

In addition, we determined the metrics of dd-cfDNA and routine biomarkers to detect biopsies with MVI as a diagnostic hallmark (AMR, mixed rejection, and DSA-MVI). (Figure 8; Table 9). The AUC-ROC of both absolute (0.88) and relative (0.88) dd-cfDNA was higher than the AUC-ROC of uACR (0.62, with higher values indicating MVI) and creatinine (0.58, with lower values indicating MVI) (all pairwise comparisons with *P* < 0.001).

Finally, DSA positivity in the absence of MVI or higher DSA-MFI was not associated with increased dd-cfDNA. Although biopsies from patients with DSA generally showed higher dd-cfDNA compared with those without DSA (n = 84, median 49; IQR 16–79 versus n = 67, median, 16; IQR 8–32; *P* < 0.001), this was not true when excluding AMR or all MVI biopsies. Dd-cfDNA was not different in DSA^+^/AMR^−^ compared with DSA^−^/AMR^−^ patients (median 17; IQR 10–54 versus median 16; IQR 8–38; *P* = 0.337) or DSA^+^/MVI^−^ compared with DSA^−^/MVI^−^ patients (median 14; IQR 10–28 versus median 13; IQR 7–25; *P* = 0.706), suggesting that the presence of DSA alone does not increase dd-cfDNA. Neither DSA-MFI at the time of biopsy nor maximum DSA-MFI at any time from first occurrence of DSA to biopsy showed significant correlation with absolute dd-cfDNA at the time of biopsy (*r* = 0.05, *P* = 0.78 and *r* = −0.03, *P* = 0.79, respectively).

## DISCUSSION

Our findings confirmed the good test metrics of dd-cfDNA in detecting rejection found in previous studies and showed that dd-cfDNA was superior to creatinine and uACR in discriminating rejection from other causes of graft dysfunction. Our data reinforce the excellent diagnostic performance of dd-cfDNA in discriminating AMR from other kidney graft pathologies and biopsies with normal findings, which is in line with earlier studies.^13-17,37^ Remarkably, dd-cfDNA was most strongly elevated in MVI, which is the hallmark feature of AMR in DSA^+^ KTR, but also of DSA-MVI or MVI of any cause, which was also demonstrated by other groups.^38-40^ Diagnoses with injury primarily occurring in the tubulointerstitial compartment, such as BKVAN, acute tubular injury, and TCMR, were also associated with higher dd-cfDNA; however, some critical aspects need to be addressed before interpreting these findings. First, dd-cfDNA did not discriminate pure TCMR excluding mixed rejection from other diagnoses. Nonetheless, in patients with TCMR biopsies showing ptc > 0 or v > 0, dd-cfDNA was above the predefined thresholds, whereas TCMR without vascular injury remained undetected by dd-cfDNA despite significant graft dysfunction leading to diagnostic biopsy (Figure 7). This distinction is highly important as it may explain the suboptimal test metrics of dd-cfDNA to discriminate borderline changes or mild TCMR from no rejection in KTR undergoing a clinical biopsy in the large DART study.^13^ Several other studies confirmed the limited capability of dd-cfDNA to detect TCMR, especially low-grade, borderline, or subclinical cases.^16,41,42^ Hence, this could translate into a relevant number of “missed rejections” if dd-cfDNA was used to rule out rejection. We consider this an important limitation for the surveillance testing with dd-cfDNA, since TCMR can have a detrimental impact on nephrons leading to late graft loss and may trigger subsequent alloimmunization, such as the development of dnDSA and AMR.^43^ Second, dd-cfDNA was clearly associated with BKVAN in our small cohort, which was described earlier.^21^ Notably, our patients with BKVAN had much higher levels of total cfDNA compared with other biopsy categories (**Table S1, SDC**, http://links.lww.com/TXD/A755) which might be a potential explanation for the low relative dd-cfDNA in BKVAN reported previously because fractional dd-cfDNA is dependent on the total cfDNA in recipients’ blood and might lead to false-negative results in cases of excessively increased total cfDNA.^17,25^ We observed this discrepancy in 2 of 6 biopsies showing BKVAN, where relative dd-cfDNA levels were low, but absolute levels were above the predefined threshold for injury, which was explained by strongly elevated total cfDNA.

We provided additional evidence for the correlation of dd-cfDNA with some Banff lesion scores, where the key lesions defining microvascular inflammation (ptc+g) showed the strongest correlation with both absolute and relative dd-cfDNA. The ptc score showed the strongest correlation with dd-cfDNA release in earlier studies, which was confirmed in our analysis.^25,37,40^ The presence and extent of ptc, especially if related to co-occurrence of DSA, had a high prognostic value for graft survival and was a pivotal contributor to transplant glomerulopathy.^44-46^ With this in mind, the ability of dd-cfDNA to reflect the presence of ptc is highly clinically important, as it allows timely diagnostic evaluation and early intervention to prevent progression to irreversible morphological alteration. Nevertheless, ptc is an unspecific lesion, seen in various pathologies, which partially explains the limited specificity of dd-cfDNA and its inability to distinguish between injury phenotypes that share the presence of ptc (AMR, DSA-MVI, TCMR, and BKVAN).^47^ Although the g-lesion showed a strong correlation with dd-cfDNA, it is more specific for MVI diagnoses per definition. However, the presence of mild glomerulitis in de novo or recurrent GN was not associated with increased dd-cfDNA release, even in cases of severe inflammation with crescent formation.^23,48^ The t-lesion showed a significant but less pronounced correlation with dd-cfDNA. This is because tubulitis was found in various diagnoses without increased dd-cfDNA levels. Isolated tubulitis (without g, ptc, or v) was associated with increased dd-cfDNA only in patients with BKVAN but not in patients with TCMR IA or IB.

Finally, absolute dd-cfDNA correlated strongly with the Banff-based AI, but only weakly with the CI. This underscores its potential to reflect real-time injury in the early-active phase of disease with greater precision than the relative dd-cfDNA associated with active and chronic lesions in this study.

Altogether, our findings support the hypothesis that dd-cfDNA circulating in peripheral blood primarily originates from the microvasculature in the graft, presumably from endothelial injury, whereas inflammatory processes predominantly affecting the tubulointerstitial compartment do not necessarily lead to dd-cfDNA release into the bloodstream. Instead, urinary biomarkers of graft inflammation, such as chemokines (eg, interferon gamma-dependent, CXCL9, and CXCL10), or alternative approaches are promising in such patterns, as end-products of tubulointerstitial damage are more likely to be captured in the urine.^49-54^

## LIMITATIONS

First, this was a single-center, observational study including indication biopsies only, which lacked an appropriate control group of “negative,” unremarkable biopsies. Therefore, an external validation cohort of dd-cfDNA-paired biopsies using the same method is desired to confirm these results.

Second, the biopsy indications were not predefined, which resulted in a more heterogeneous cohort, and consequently, higher variability in diagnoses. This makes the study results explorative, on the other hand provides more “real-world” findings as compared with other studies. Third, granular grouping of diagnosis based on histopathological and clinical findings may limit generalizability. Fourth, most biopsies were performed in the later posttransplant period, with a median of 7.5 y after transplant. This resulted in a lower frequency of TCMR than AMR, which limits the generalizability of the findings on TCMR. Finally, electron microscopy was only available for 51% of the biopsies, and 13% of the biopsies did not meet the Banff criteria for marginal specimen adequacy of ≥7 glomeruli and ≥1 artery.

## CONCLUSIONS

We conclude that blood dd-cfDNA is an excellent biomarker of microvascular inflammation but remains an imperfect rejection biomarker. We suggest a risk-stratified approach, such as screening for AMR in patients with high immunological risk (eg, DSA-positive), which can enhance the diagnostic value of dd-cfDNA and provide additional information in ambiguous clinical settings.^55,56^ To fully validate the clinical utility of this biomarker in routine practice, future studies should incorporate clinical endpoints to demonstrate the value of this promising biomarker in improving graft survival or other patient-centered outcomes.

## ACKNOWLEDGMENTS

The authors wish to thank all the patients for their participation and contribution to this study.

## Supplementary Material


