# Supplementary material for: Association of Blood Donor-derived Cell-free DNA Levels With Banff Scores and Histopathological Lesions in Kidney Allograft Biopsies: Results From an Observational Study
Source: Transplant Direct. 2025 Apr 10;11(5):e1794. doi: 10.1097/TXD.0000000000001794 (PMC12313090; doi:10.1097/TXD.0000000000001794)
Supplement: Supplementary file 1 [file txd-11-e1794-s001.pdf]

Supplemental Digital Content (SDC)

SDC, Material and methods.

Detailed description of droplet-digital-PCR (ddPCR)-based quantification method of dd-cfDNA.

For each patient, four informative single nucleotide polymorphisms (SNPs), defined as SNPs for which the recipient is homozygous, and the donor carries at least one heterozygous allele, were selected from a set of 40 predefined SNPs. These four SNPs were used to quantify the dd-cfDNA concentration (%) as: donor alleles/ (donor alleles + recipient alleles). The results for SNPs with heterozygous donor genotypes were corrected by a factor of 2. Total cfDNA was extracted from up to 8 mL of plasma, and the concentration was determined as haploid genome copies using droplet-digital PCR and the result was corrected for extraction loss and cfDNA fragmentation, as described previously.[S1] The absolute concentration of dd-cfDNA (copies/mL plasma) was calculated by multiplying total cfDNA (copies/mL) and dd-cfDNA (%). Reference ranges for total cfDNA in the post-transplant course were previously established in a cohort of 300 KTR.[S2] Absolute quantification in contrast to fractional determination (%) has the advantage that is it not affected by changes in recipient cfDNA, for example by infection. Changes in total cell-free DNA due to leukopenia or leucocytosis can alter dd-cfDNA fraction (%) leading to false positive or negative results. Absolute quantification is not affected. [S2, S3]

SDC, Table S1. Total recipient cfDNA values according to different histological diagnoses.

**Histological diagnosis (n)**	**total recipient cfDNA (median, cp/mL)**
ABMR (45)	4210 (2731 - 7721)
aABMR (7)	3335 (1831 - 5873)
caABMR (38)	4337 (2858 – 8447)
Mixed Rejection (TCMR+ABMR) (8)	5579 (5006 - 8635)
DSAnegMVI (6)	4317 (3043 - 13886)
TCMR (11)	7726 (4918 -9341)
BKVAN (6)	14039 (2526 – 16068)
CNI-Toxicity (19)	2602 (1953 – 3154)
Glomerulonephritis (20)	4465 (2526 – 6230)
UTI (6)	2909 (2188 – 3432)
IFTA (12)	4570 (3576 – 5096)
Other (12)	6268 (5012 – 10581)

Normal (6)	3165 (2197 – 4822)

SDC References:

1. Beck J, Bierau S, Balzer S, Andag R, Kanzow P, Schmitz J, Gaedcke J, Moerer O, Slotta JE, Walson P, Kollmar O, Oellerich M, Schütz E. Digital droplet PCR for rapid quantification of donor DNA in the circulation of transplant recipients as a potential universal biomarker of graft injury. Clin Chem. 2013 Dec;59(12):1732-41. doi: 10.1373/clinchem.2013.210328. Epub 2013 Sep 23.

2. Oellerich M, Shipkova M, Asendorf T, Walson PD, Schauerte V, Mettenmeyer N, Kabakchiev M, Hasche G, Gröne HJ, Friede T, Wieland E, Schwenger V, Schütz E, Beck J. Absolute quantification of donor-derived cell-free DNA as a marker of rejection and graft injury in kidney transplantation: Results from a prospective observational study. Am J Transplant. 2019 Nov;19(11):3087-3099. doi: 10.1111/ajt.15416. Epub 2019 May 28.

3. Osmanodja B, Akifova A, Budde K, Choi M, Oellerich M, Schütz E, Beck J. Absolute or Relative Quantification of Donor-derived Cell-free DNA in Kidney Transplant Recipients: Case Series. Transplant Direct. 2021 Oct 22;7(11):e778. doi: 10.1097/TXD.0000000000001237.
